# Effectiveness of Telehealth in Rural and Remote Emergency Departments: Systematic Review

**DOI:** 10.2196/30632

**Published:** 2021-11-26

**Authors:** Christina Tsou, Suzanne Robinson, James Boyd, Andrew Jamieson, Robert Blakeman, Justin Yeung, Josephine McDonnell, Stephanie Waters, Kylie Bosich, Delia Hendrie

**Affiliations:** 1 School of Population Health Curtin Univeristy Bentley Australia; 2 Innovation & Development WA Country Health Service Perth Australia; 3 Digital Health La Trobe University Bundoora Australia; 4 Consumer and Community Health Research Network Nedlands Australia; 5 Consumer and Mental Health WA Cloverdale Australia; 6 Command Centre WA Country Health Service Perth Australia

**Keywords:** telehealth, telemedicine, clinical effectiveness, treatment outcome, rural population, rural health, remote

## Abstract

**Background:**

Emergency telehealth has been used to improve access of patients residing in rural and remote areas to specialist care in the hope of mitigating the significant health disparities that they experience. Patient disposition decisions in rural and remote emergency departments (EDs) can be complex and largely dependent on the expertise and experience available at local (receiving-end) hospitals. Although there has been some synthesis of evidence of the effectiveness of emergency telehealth in clinical practice in rural and remote EDs for nonacute presentations, there has been limited evaluation of the influence of contextual factors such as clinical area and acuity of presentation on these findings.

**Objective:**

The aims of this systematic review are to examine the outcome measures used in studying the effectiveness of telehealth in rural and remote EDs and to analyze the clinical context in which these outcome measures were used and interpreted.

**Methods:**

The search strategy used Medical Subject Headings and equivalent lists of subject descriptors to find articles covering 4 key domains: telehealth or telemedicine, EDs, effectiveness, and rural and remote. Studies were selected using the Population, Intervention, Comparator, Outcomes of Interest, and Study Design framework. This search strategy was applied to MEDLINE (Ovid), Cochrane Library, Scopus, CINAHL, ProQuest, and EconLit, as well as the Centre for Reviews and Dissemination databases (eg, National Health Service Economic Evaluation Database) for the search period from January 1, 1990, to May 23, 2020. Qualitative synthesis was performed on the outcome measures used in the included studies, in particular the clinical contexts within which they were interpreted.

**Results:**

A total of 21 full-text articles were included for qualitative analysis. Telehealth use in rural and remote EDs demonstrated effectiveness in achieving improved or equivalent clinical effectiveness, appropriate care processes, and—depending on the context—improvement in speed of care, as well as favorable service use patterns. The definition of effectiveness varied across the clinical areas and contexts of the studies, and different measures have been used to affirm the safety and clinical effectiveness of telehealth in rural and remote EDs. The acuity of patient presentation emerged as a dominant consideration in the interpretation of interlinking time-sensitive clinical effectiveness and patient disposition measures such as transfer and discharge rates, local hospital admission, length of stay, and ED length of stay. These, together with clinical area and acuity of presentation, are the outcome determination criteria that emerged from this review.

**Conclusions:**

Emergency telehealth studies typically use multiple outcome measures to determine the effectiveness of the services. The outcome determination criteria that emerged from this analysis are useful when defining the favorable direction for each outcome measure of interest. The findings of this review have implications for emergency telehealth service design and policies.

**Trial Registration:**

PROSPERO CRD42019145903; https://tinyurl.com/ndmkr8ry

## Introduction

### Background

The significant health disparities for residents of rural and remote communities compared with metropolitan or urban populations raise questions of equity and access to health services. Multiple reasons have been put forward internationally to explain these health disparities [1-4]. Limited access to health care is seen as a major contributor to rural or remote and metropolitan or urban health disparities, with workforce supply central to this discourse [5-10].

Emergency department (ED) services are an essential component of the health system, often serving as the first or only point of contact for patients requiring medical care. Patients presenting to tertiary center EDs, often in an urban location, can be assured of a well-supported ED with sufficient post-ED care within the same hospital or at another hospital within a short distance [11]. Transfer decisions in rural and remote EDs do not generally have the assurance of timely and appropriate follow-up care. The challenges in rural and remote ED care are 2-fold. First, variations are evident in the capability of local hospitals, arising mainly from the lack of economy of scale to justify investments in a full range of capabilities and inpatient wards for continued treatment and monitoring after completion of ED care. Second, the time needed and distance involved to reach definitive health care compared with suburban or urban settings [12] can delay time-critical treatments such as thrombolytic treatments to resolve a dangerous clot in the blood vessels. This means that, in making decisions around patient dispositions, local hospital capabilities and distance from destination hospitals should be considered together with the patient’s clinical conditions.

Emergency telehealth services provide rural and remote hospitals with timely specialist expertise to increase staff support during critical ED encounters [12], to some extent mitigating the inequities in workforce supply. The key question in the evaluation of the effectiveness of an emergency telehealth service is whether this increased specialist workforce participation in rural and remote ED presentations improves patient outcomes by delivering more timely and effective care.

Only 1 systematic review has examined the use of telehealth in rural and remote EDs, and its focus was on noncritical emergency presentations [11]. The scope of ED services included in the review ranged from telepsychiatry to assist with mental health emergency presentations and teleophthalmology for acute eye concerns requiring ophthalmologist assessment to tele-emergency, half of which involved the use of teleradiology and consultation with other subspecialists [11]. The outcomes of interest were uptake of the telehealth program, change in diagnosis or management plan, patient transfer rate, and patient dispositions (discharge, local admission, and discharge against medical advice) [11]. Of the 15 studies reviewed, 5 reported the influence of telehealth on patient diagnosis or management, with teleconsultations changing the diagnosis or management in 18%-66% of the consultations [13-17]. The review also discussed the dependence of patient dispositions on telehealth program design and observed close linkages between the rate of patient transfer, discharge, local admission, and discharge against medical advice and emergency telehealth use [11]. Most of the studies included in the review reported increases in patient transfer rates [11]. A total of 4 studies aligned telehealth with a reduction in unnecessary patient transfers [15,18-20]. Apparent in the review was the reduction of unnecessary transfers and secondary overtriage (misidentification of noncritical patients as critically ill at initial presentation), which translated into increased local hospital admissions and reduced discharge after teleconsultation [19,20].

Whether reduced unnecessary transfer and increased local admission are favorable outcomes for patients depends on the acuity and health conditions being treated as well as the infrastructure and workforce capabilities of the local hospitals to accommodate the increased demand. For the same reasons, transfer avoidance may not always result in favorable outcomes for patients presenting to rural and remote EDs. Similarly, an increase in local hospital admission may not always lead to favorable patient outcomes if specialist consultations through telehealth alone do not change the capability of local (receiving-end) hospitals to continue caring for patients presenting to the ED in critical conditions. While identifying outcome measures, the systematic review of the use of telehealth in managing emergencies in rural and remote EDs did not consider the relevance of outcome measures across the various clinical contexts such as clinical area and the acuity of presentation. In addition, its focus was on noncritical presentations only [11].

### Objective

The aim of this systematic review is to examine the outcome measures used in studying the effectiveness of rural and remote emergency telehealth services and analyze the clinical context in which these outcome measures were used and interpreted. The findings from this review provide insight into evaluating the clinical impact of telehealth services in rural and remote EDs and will assist in the design of future studies on the effectiveness and cost-effectiveness of emergency telehealth services in the rural and remote context.

## Methods

### Study Selection

This systematic review followed the effectiveness part of a published protocol on reviewing the literature on the effectiveness and cost-effectiveness of telehealth services in rural and remote EDs [21]. Studies were selected using the Population, Intervention, Comparator, Outcomes of Interest, and Study Design framework (see Table 1 for inclusion and exclusion criteria). Although there was a substantial body of literature on using telehealth to support prehospital emergency medical services, to restrict the scope, this review only included studies taking place in hospital EDs and excluded records reporting on the use of prehospital emergency telehealth.

**Table 1:** Selection criteria.

Parameter	Inclusion criteria	Exclusion criteria
Population	Rural and remote populations treated in emergency departments	Rural and remote populations treated outside of the emergency department
Intervention and comparator	Emergency telehealth versus treatment as usual including the following: Telephone versus face-to-face consults Videoconference versus telephone consults	Descriptive studies without comparators Study focused on a mobile device or electronic health records
Outcomes	Timeliness of care Health service use Clinical effectiveness	Descriptive statistics without a well-defined effectiveness measure
Study design	Randomized controlled trials Nonrandomized controlled trials Qualitative research	Commentaries Expert opinions Government reports Strategic documents Single-case reports

### Information Sources and Search Strategy

**Figure 1:**
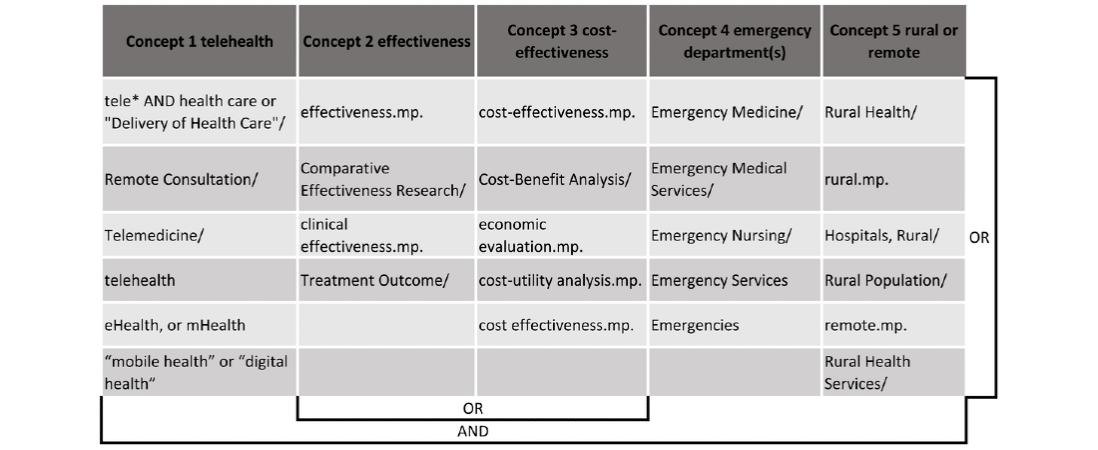
Search strategy.

The search strategy used Medical Subject Headings and equivalent lists of subject descriptors to find articles covering 4 key domains: telehealth or telemedicine, EDs, effectiveness, and rural and remote (Figure 1).

This search strategy was applied to MEDLINE (Ovid), Cochrane Library, Scopus, CINAHL, ProQuest, and EconLit, as well as the Centre for Reviews and Dissemination databases (eg, the National Health Service Economic Evaluation database) for the search period from January 1, 1990, to May 23, 2020. The reference lists of the included studies were hand searched to include other peer-reviewed publications relevant to this review. Finally, a search was conducted on Google using the phrase “effectiveness of rural and remote emergency department telehealth.”

### Study Records

#### Data Management and Selection Process

The identified records were downloaded into EndNote (Clarivate), where duplicate records were identified and removed. A reviewer (CT) screened all the downloaded titles and excluded those that were irrelevant to the review. The abstracts of the preliminary list were classified into included, excluded, and *gray area* according to the study selection criteria [21]. The *gray area* abstract entries were independently reviewed by 1 of the 3 other reviewers (SR, DH, or JB). The articles excluded at full-text review were reviewed by another reviewer. Data extraction was organized into data collection tables, which were checked by a second reviewer (SR, DH, or JB). Any disagreements were reviewed by a third reviewer and agreed upon through discussion. The Joanna Briggs Appraisal Checklists [22] that corresponded to the study designs were used to assess the quality of the studies included for detailed review. Alignment to more than 75% of all checklist items was considered high-quality reporting, alignment to between 50% and 74% was considered moderate-quality reporting, and alignment to less than 50% was considered poor-quality reporting [23,24].

#### Data Extraction and Synthesis

**Figure 2:**
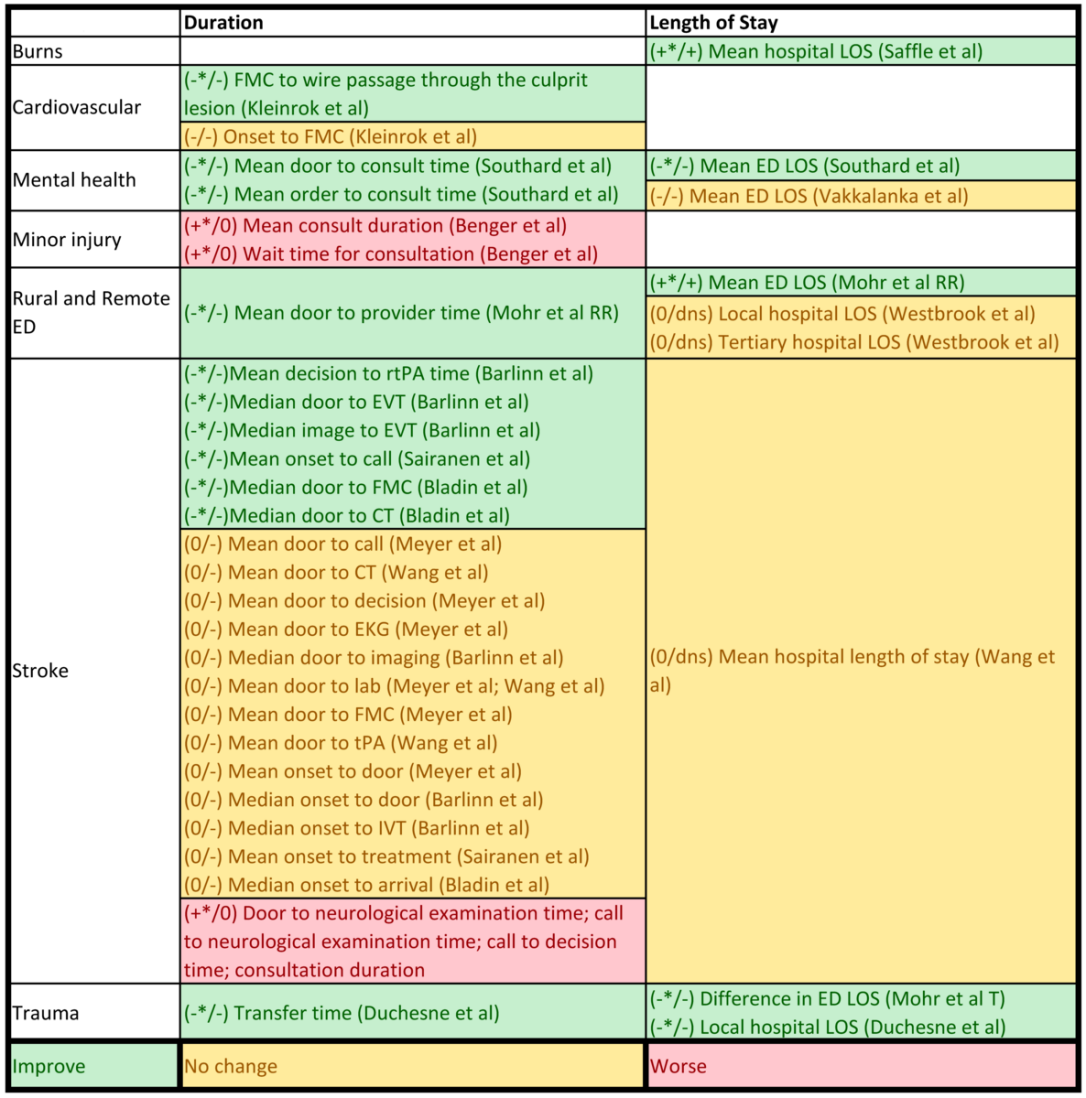
Summary of time-sensitive clinical effectiveness.

**Figure 3:**
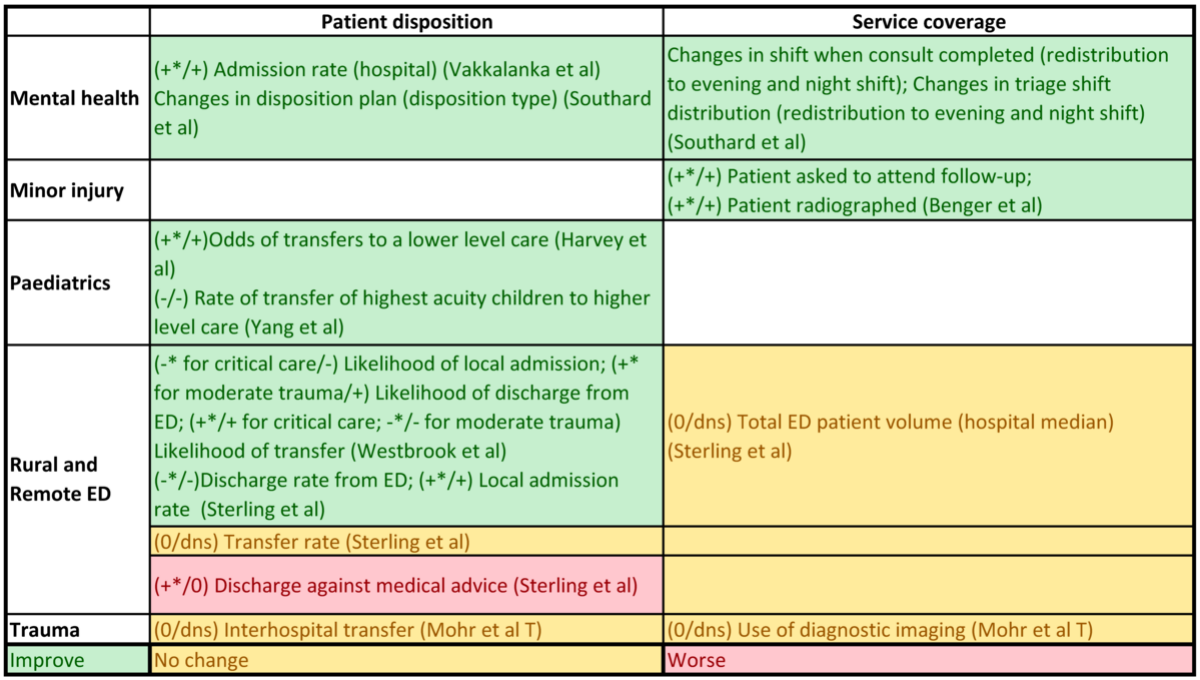
Summary of health service use measures.

**Figure 4:**
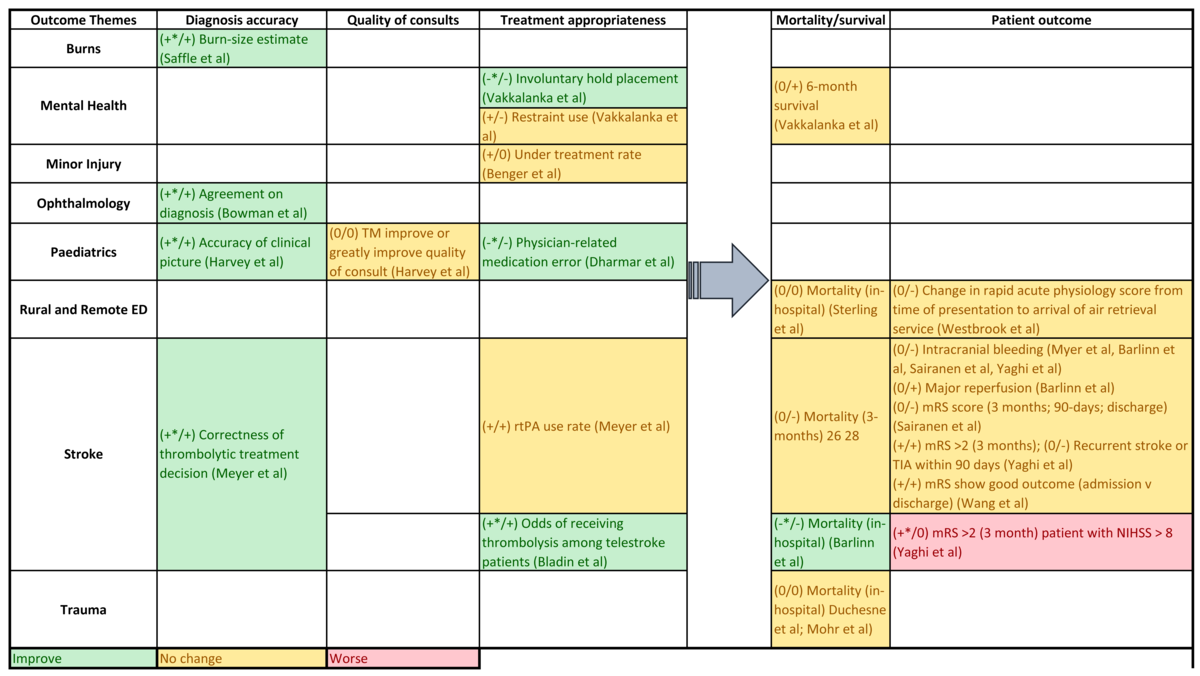
Summary of clinical effectiveness.

The articles included for full-text review were categorized by clinical area, country, and operational use of telehealth interventions (whether the telehealth was used to provide direct consultation to patients at a remote end, to support local clinicians in face-to-face patient care, or for remote monitoring of changing health conditions). The outcome measures from each study were also mapped against the clinical areas to understand the use context of each type of measure. Each of the study outcomes was separately reviewed by categorizing them into clinical effectiveness or service use measures, the context in which the outcomes were used, and any validity or data quality issues noted. Data from the effectiveness studies summarized above were used to build an evidence table for each of the outcome measures identified. Salient trends were extracted from this evidence table to compile separate summaries on outcome measure use with favorable directions of change for the time-sensitive clinical effectiveness (Figure 2), service use (Figure 3), and clinical effectiveness measures (Figure 4).

## Results

### Study Selection

**Figure 5:**
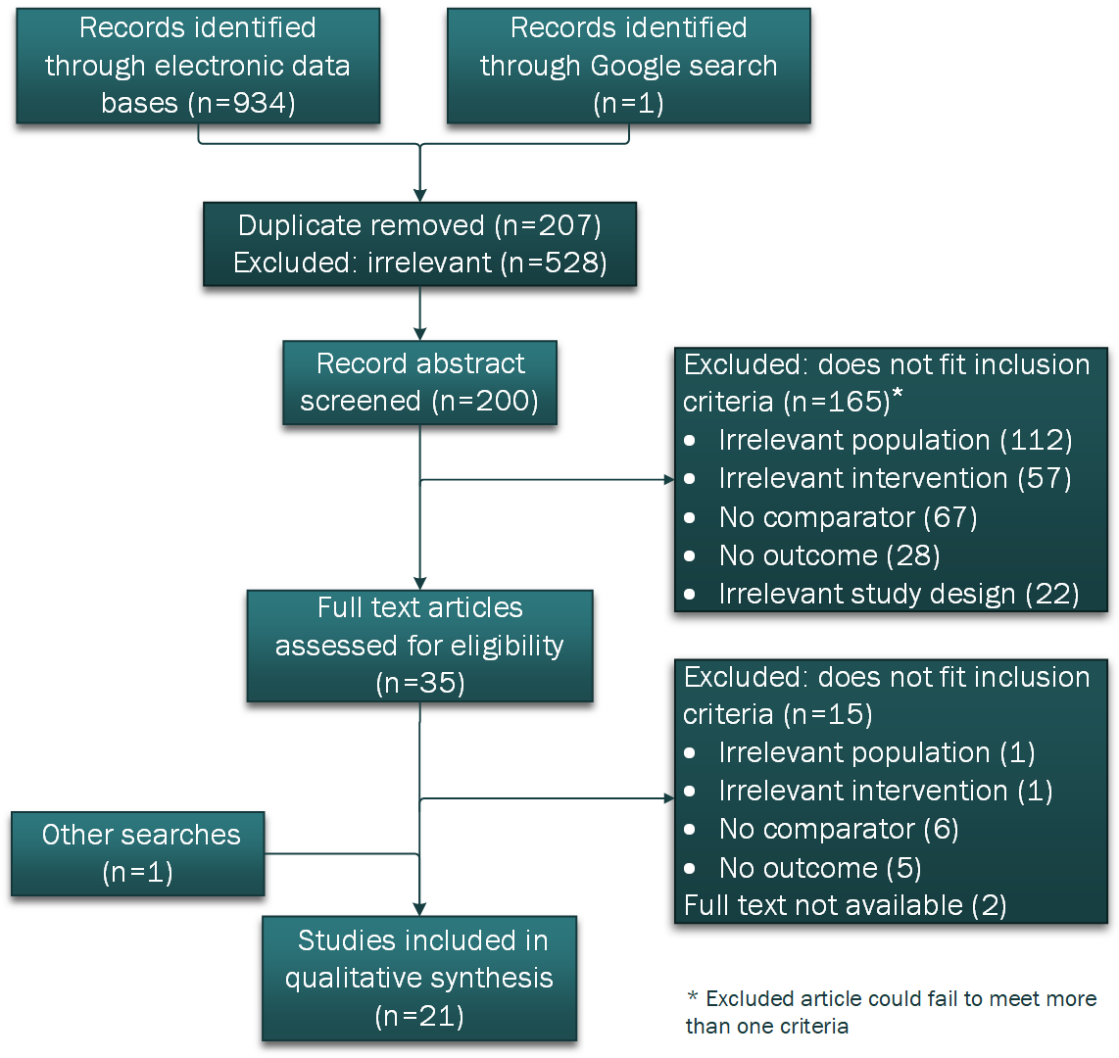
Flow chart diagram of the study selection.

The search of the electronic databases identified 934 records for title screening. An additional record was identified from the Google search. Of the total 935 records, 207 (22.1%) duplicate titles and 528 (56.5%) irrelevant titles were removed. Of the remaining 200 abstracts screened, 165 (82.5%) were excluded because they did not fit 1 or more of the inclusion criteria. A full-text review of the remaining 35 records identified a further 15 (43%) that did not meet 1 of the inclusion criteria. An additional record was identified from hand searching of the reference lists of the included records. Detailed review and data extraction were performed on 21 articles. A PRISMA (Preferred Reporting Items for Systematic Reviews and Meta-Analyses) flow diagram is presented in Figure 5.

Multimedia Appendix 1 [25-36] summarizes the bibliographic information from individual records, including information related to the telehealth intervention, study details, and the authors’ assessment of the reporting quality. Of the 21 studies, 14 (67%) were of high-quality reporting according to the criteria used in this review.

### Study Characteristics

The studies were categorized according to clinical area and country of implementation (Table S1 of Multimedia Appendix 2 [25-36]). Of the 21 records reviewed, the most frequently assessed service (6/21, 29%) was telestroke [16,25-28,36]. Other clinical area categories included ED services in rural and remote regions [12,19,20,29], trauma [30-32], pediatrics [15,33], mental health [18,34], ophthalmology [14], minor injury [13], cardiovascular [35], and burns [17]. Of the 21 studies, 13 (62%) reported telehealth interventions implemented in the United States [12,15-18,20,27-29,31-34]; the other countries reporting on telehealth interventions were the United Kingdom [13,14], Australia [19,36], Finland [26], Germany [25], Norway [30], and Poland [35].

A further categorization was conducted of the clinical area by operational use of telehealth (Table S2 of Multimedia Appendix 2 [25-36]). Of the 21 studies, 12 (57%) involved a specialist supporting local clinicians [12,14,15,17,19,25-28,33,34,36], 8 (38%) involved a direct consultation by a specialist physician [13,16,18,20,29-32], and 1 (5%) concerned the provision of diagnostic services [35]. Of the 8 direct consultations, 7 (88%) were to a location staffed by a nurse practitioner, whereas 1 (12%) [18] involved a mental health specialist directly consulting the patient in the absence of a local clinician. No study documented an intervention involving remote monitoring in the rural and remote emergency setting.

### Study Designs of Included Studies

**Figure 6:**
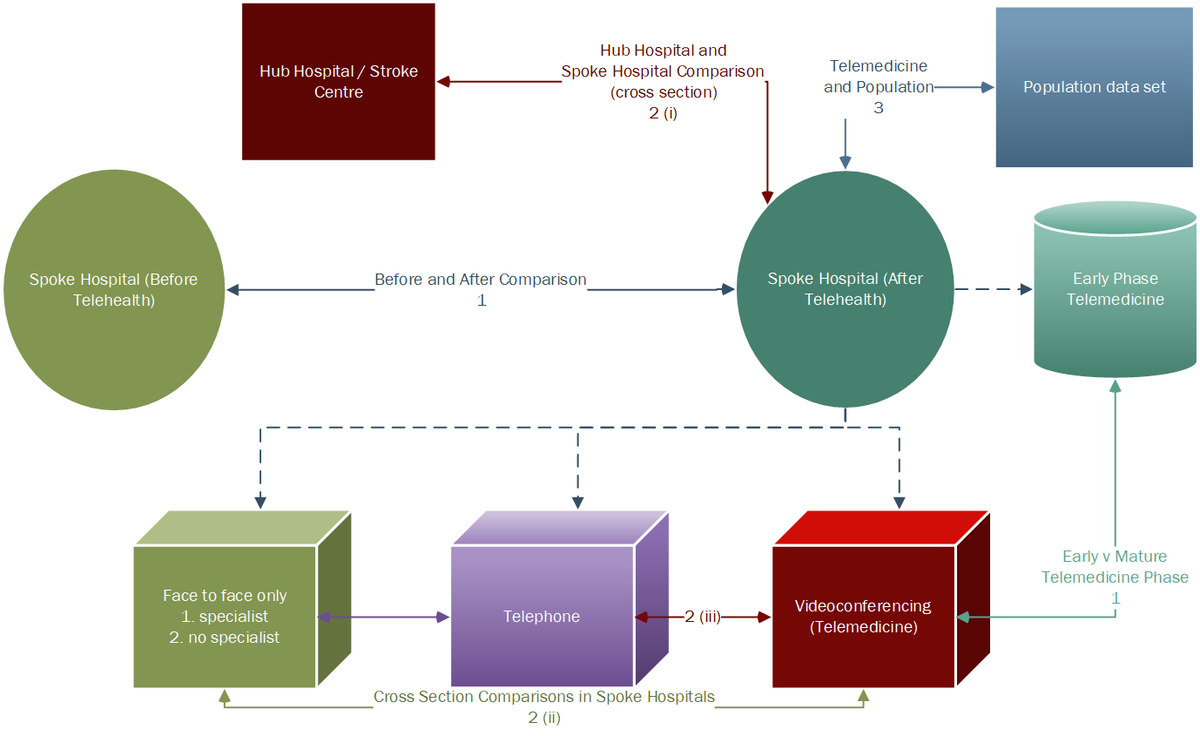
Comparisons used in rural and remote emergency department telehealth studies.

The studies included in this review identified study cohorts according to whether the patients had telephone, videoconferencing, or face-to-face consultations. In terms of the study design, 14% (3/21) of the studies were intervention studies—of these 3 studies, 2 (67%) were randomized [13,16] and 1 (33%) was nonrandomized [14])—10% (2/21) were qualitative studies [12,30], and 76% (16/21) were cohort studies [15,17-20,25-29,31-36]. There were 3 types of comparisons: (1) comparing before-and-after telehealth interventions at spoke hospitals [12,17-20,29,31] (the comparison of early-phase telemedicine implementation with the mature phase of telemedicine implementation in Kleinrok et al [35] and Bladin et al [36] is also a form of before-and-after comparison); (2) cross-sectional comparisons such as the following: (i) comparison between the videoconferencing consultation (typically referred to as telemedicine) at the spoke hospital and face-to-face consultation at the hub hospital [25,27,28], (ii) comparison within spoke hospitals between cohorts of patients who received telemedicine consultation and patients receiving only face-to-face consultations with a local clinician [13,14,32-34], and (iii) comparison between telemedicine and telephone consultations [15,16,30,33]; and (3) comparison of a telemedicine study cohort with population cohort–based data. A study on patients who had received thrombolysis compared the study group with population thrombolysis data sets [26]. Table S3 of Multimedia Appendix 2 [25-36] lists author names and year of publication against the study design categories and comparison categories. Figure 6 illustrates the different types of comparisons.

There was variability among the hub-and-spoke comparison studies, all of which were related to telestroke. The focus of 10% (2/21) of the studies was on patient disposition after thrombolysis: Wang et al [27] compared patients who received thrombolysis locally with patients who were transferred and then received thrombolysis at the hub, whereas Yaghi et al [28] compared the data of patients who had received thrombolysis locally, were transferred to the hub, and stayed locally. Barlinn et al [25] compared patients who had had a stroke and who had been transferred to the hub after telestroke consultation with patients who had been admitted directly to the hub.

### Synthesis of Effectiveness Findings

#### Overview

The included quantitative studies typically used multiple measures within a study to assess effectiveness. Of the 19 quantitative studies, 13 (68%) showed improvement in 1 of the primary outcomes [14-20,27-29,31-35] and 6 (32%) in 1 of the secondary outcomes [13,15,17,18,29,34]. The effectiveness measures were categorized into time-sensitive effectiveness, service use measures, and clinical effectiveness, including patient outcome.

#### Time-Sensitive Effectiveness Measures

##### Overview

Of the 19 quantitative studies, 11 (57%) showed improvement in time-sensitive clinical effectiveness, including significant changes in ED length of stay [18,29,32], hospital length of stay [17,31], and reduction in duration of ED care processes (increased speed of care) [18,25,26,29,31,35,36]. Figure 2 summarizes the direction and findings on length of stay and care process measures by clinical area.

##### Length of Stay

Length of stay was a measure of clinical effectiveness used in the studies, and this has direct implication on resource use. However, a decreased length of stay was not always interpreted as a favorable outcome across all studies. Increased hospital length of stay at the burn center was used to indicate a higher degree of diagnostic accuracy at the local hospital [17], whereas videoconferencing consultation was shown to improve the accuracy of triage [17]. Decreased local hospital length of stay was considered favorable in trauma care because of the promptness of radiologic evaluation through telehealth intervention [31], leading to faster disposition decisions. Similarly, a reduction in the ED length of stay reflected timely ED care for trauma [32] and mental health [18,34], whereas a longer ED length of stay reflected the expanded capacity of local EDs to manage patients locally [29]. Of the 19 studies, 2 (10%) showed equivalent local and tertiary hospital lengths of stay [19,27]; no study showed a worse outcome in terms of length of stay.

##### Care Processes

The process-of-care measures have been used to indicate service efficiency, but they are not always related to effectiveness. Improvements in care processes were shown in cardiovascular, stroke, and trauma care and were related to reaching clinical decisions and providing treatment interventions faster than usual care [16,25,26,31,35]. A significant reduction in door-to-consultation time was noted in delivering specialist mental health services [18] and when a combined rural and remote ED cohort [29] was studied. Treatment-related duration can affect clinical effectiveness in some clinical areas, for example, in the case of telestroke, time to thrombolysis (recombinant tissue plasminogen activator) or door-to–endovascular thrombectomy time. None of the studies provided direct evidence on the relationship between treatment-related durations and patient outcome.

Telehealth in rural and remote EDs made little difference to speed of care when the time gap was calculated from the point of symptom onset [16,25-27,35,36]. In the context of stroke care, earlier studies demonstrated that involving telehealth did not delay or speed up time to imaging and laboratory tests [16,25,27]. This was likely to be related to the reliance of stroke diagnosis on imaging and pre-established clinical standards in practice. However, an Australian study has shown results to the contrary where telestroke resulted in shorter door-to–computed tomography scan time and door-to-needle time for stroke thrombolysis [36]. A telestroke study showed longer time to receiving a neurology examination and reaching a clinical decision when videoconferencing was compared with telephone consultations [16]. Consult durations were longer in the clinical areas of minor injury than in face-to-face specialist consultations and among patients who had had a stroke and who received thrombolysis [13,26]; however, the duration of the consult decreased as health services became acquainted with the telehealth technology. These studies did not discuss the significance of this finding; however, a 2- to 10-minute difference in consult duration was minimal, considering the improved accuracy and appropriateness of the transfer decisions.

##### Health Services Use Measures

Of the 19 quantitative studies, 6 (32%) showed significant improvement in service use patterns in the clinical areas of rural and remote EDs [19,20], mental health [18,34], minor injury [13], and pediatrics [15]. In all, 5 of the service use measures were related to patient disposition, including significant change in hospital admissions [19,20,34], rate of discharge from ED [19,20], appropriateness of transfer [15,19], and changes in disposition plan [18], whereas 2 were related to significant changes in service coverage, including an increasing proportion of out-of-hour triage and consultation [18] and the proportion of patients asked to attend follow-up clinics [13]. The direction of outcome deemed favorable depended on the context of the study. Figure 3 summarizes patient disposition and service coverage measures and findings from the included studies by clinical area.

##### Transfer Rates

Whether to transfer a patient and to which location are important clinical decisions in rural and remote EDs. Depending on the capability and capacity of local hospitals, the acuity of presentation and the level of definitive care sought by the transfer are directly related to the decisions to transfer or stay locally. Transfer rates were interpreted together with admission and discharge rates and had the function of examining the appropriateness of service use. An acuity subgroup analysis in Westbrook et al [19] demonstrated significant variation in transfer rates between critical care and moderate trauma. That is, telehealth increases the transfer rate of patients classified as high acuity, which is mirrored in the reduced likelihood of local admission in this cohort. Similarly, the significant reduction in transfer likelihood of patients with moderate trauma is reflected in the increased likelihood of discharge from local EDs [37]. Pediatric emergency telehealth studies reported increased odds of transfer to lower-level care in pediatric triage [15] and reduced transfer in pediatric patients with the highest-acuity ED presentations compared with telephone consultations [38]. A study interpreted the observations on rates of transfer together with the clinicians’ subjective perception of increased accuracy of the clinical picture before arrival at the tertiary hospital [15], whereas another study regarded changes in transfer rates as an indication of health services use appropriateness [19].

##### Local Admissions and Discharge From ED

Patients who are not transferred are either admitted locally or discharged home. This is another decision made by ED clinicians in consultation with emergency medicine specialists through telehealth. The reduction in the rates of discharge from local EDs and increased local admission was observed in Sterling et al [20] after the implementation of emergency medicine specialist consultation with a local nurse practitioner. This study did not stratify by acuity of presentation, and the higher rate of local admission explained the decreased ED discharge rate [20]. The change in discharge rate accompanying increased local admission is contrary to that reported in Westbrook et al [19], which involved decision-making by local ED physicians in consultation with emergency medicine specialists.

A reduced likelihood of local admission for patients classified as critical care and increased likelihood of discharge from the ED for patients with moderate trauma in Westbrook et al [19] indicated the effectiveness of the intervention in identifying patients who did not require further care, whereas in Sterling et al [20], increased local admission rate and decreased rate of discharge from the ED were used to reflect the benefit of telemedicine in augmenting local capacity to care for patients locally, and this translated into increased financial viability of local hospitals. The patients in Westbrook et al [19] were classified as triage category 1 or were those with major or moderate trauma, skewing toward higher acuity compared with the Sterling et al [20] patient cohort. The variation in patient acuity reflected the base local hospital capability that the emergency medicine specialists were supporting. This demonstrated the differing use of these measures across patient acuity levels.

In addition, the different patterns of changes in the rates of transfer and discharge from the ED between the Westbrook et al [19] and Sterling et al [20] studies are not likely to be attributable to telehealth consultations. It is more likely a result of the difference in disposition practices for patients who stayed locally. Spoke hospitals in Westbrook et al [19] may have cared for and discharged patients from EDs, whereas in Sterling et al [20], the patients may have been transferred to the inpatient department for the same care, which is why they were not considered discharged from the ED.

For mental health ED presentations, an increased combined rate of hospital admissions [34] and an increased range of dispositions [18] were considered a favorable outcome from telehealth interventions. Sterling et al [20] showed an increased rate of discharge against medical advice after implementation of a telehealth service. In other words, when the treating physician was not physically present, patients were more likely to act against medical advice.

##### Service Coverage

Telemedicine was effective in redistributing or increasing service coverage in the ED. Southard et al [18] demonstrated a redistribution of mental health triage and consultation completed in the evening and night shifts, and Benger et al [13] showed an increase in the proportion of patients with minor injuries asked to attend follow-up appointments after telemedicine consultation. Other service coverage measures included the proportion of patients radiographed for minor injury [13], median total ED patient volume [20], and the odds of using diagnostic imaging in patients with trauma [32], but the studies did not show significant change in these service coverage measures.

#### Clinical Effectiveness Measures

Clinical effectiveness is an indication of safety and quality and can be used as a surrogate measure for patient outcome. Of the 19 quantitative studies, 9 (47%) showed improvement in clinical effectiveness, including diagnosis accuracy [14,15,17], treatment appropriateness [16,33,34], and improved patient outcome [25,27,28], whereas 15 (71%) showed that telemedicine can achieve effectiveness similar to that achieved by the comparator interventions. The effectiveness measures included in-hospital mortality [20,31,32,36], 3-month [26,28] and 6-month [34] mortality, treatment complications [16,25,26,28,36], patient outcomes [16,19,25-28], treatment rates [13,16,34], and consult quality [15].

The measures for diagnosis accuracy and patient outcome were clinical area dependent. A generalized study on telehealth in rural and remote EDs used change in the rapid acute physiology score from time of presentation to arrival of air retrieval service as a measure of stability among transferred patients [19]. Apart from the rapid acute physiology score, all other patient outcome measures were stroke related (ie, functional scores, major reperfusion after thrombolysis, intracranial hemorrhage, and recurrent stroke rates) [16,25-28]. Figure 4 summarizes the categories of the clinical effectiveness measure by clinical area, with suggested outcomes hierarchy among these measures. As reported above, treatment-related, time-sensitive clinical effectiveness measures can also be surrogate measures for patient outcomes.

### Risk of Bias in Individual Studies

The most significant risk of bias within the studies was related to study design. The risk of bias is high in before-and-after comparison studies because systems change over time, which cannot be controlled [20]. The studies did not report on the details of system change; therefore, this risk of bias could not be addressed in this review. Study designs involving cross-sectional comparisons could not be randomized and experienced selection bias pertaining to severity, with higher severity among the telemedicine groups, that is, tendency to consult emergency medicine specialists when managing patients classified as more severe.

The tendency to recruit greater number of telemedicine cases from larger spoke hospitals diluted the effect of remoteness on the effectiveness of telehealth when the results were interpreted as a whole. This is a significant selection bias in telehealth studies and highlights the gap in the published literature on the impact of telehealth in remote and very remote regions.

Other risks of bias in individual studies related to the sample sizes. Of the 21 studies, 7 (33%) had sample sizes of fewer than 100 patients [14,16-19,26,27]. The studies also reported selection bias in relation to local (receiving-end) clinicians’ preselected cases for telehealth consultations when hospital transfers were considered. This process selected patients whose transfers were more imminent after telemedicine specialist consultations.

A further weakness in many of the included studies was that of attribution. The studies assumed the clinical or service use effectiveness to be associated with the telehealth interventions without considering potential confounders.

## Discussion

### Principal Findings

#### Context of Intervention

Telehealth interventions were considered effective when their implementation resulted in improvement or equivalent clinical or service use outcomes. The indicators used to measure favorable patient outcome were unidirectional; that is, better outcome pertains to change of the indicator in 1 direction. However, time-sensitive effectiveness and service use measures were interpreted differently, depending on the context of the intervention.

#### Patient Dispositions

Patient disposition measures were dependent on the severity of the presenting illness and the level of definitive care compared with the hospital of origin and clinical area. Higher rate of transfer and shorter local ED or hospital length of stay were considered favorable in higher acuity ED presentations such as triage 1 or major trauma, and the reverse was true in less life-threatening conditions such as mental health and moderate trauma. This concurs with the findings from the observational study conducted on the North Dakota critical-access hospital ED cohorts where the interhospital transfer rate was not associated with telehealth use after adjusting for severity of illness [39]. The pediatric emergency telehealth studies considered an additional factor of the relative level of definitive care. Transfers to lower-level care increased with telehealth consultations [15], whereas transfers to higher-level care decreased in pediatric cases with the highest acuity [38].

#### Timeliness of Care Measures

Telestroke was effective in maintaining the same level of timeliness once a patient arrived at the ED, whereas in other clinical areas, telehealth resulted in faster transitions from consultation to diagnosis and treatment. Timeliness of the care measures reflected factors modifiable by telehealth. For example, the significant reduction of door-to-consultation time may be explained by the shortage of workforce and specialist skills in the rural and remote EDs, which were modifiable using telehealth consultations. The observation in telestroke—no significant change in timeliness of care—was explained by the dependence on access to imaging and other diagnostic tests, which were local processes that were not modifiable by telehealth consultations. The competent use of technology was also a factor influencing time-sensitive clinical effectiveness.

#### Changing Service Use Patterns

Favorable service use patterns involved a redistribution of resources, such as changes in disposition plan, changes in the pattern of interhospital transfers [19,20,32,37,38], or redistribution of ED triage and medical consultation to evening and night shifts instead of a concentration of medical consultations during day shifts [18]. These changes indicated the effectiveness of telehealth in facilitating more appropriate use of health resources, which did not always translate into an absolute reduction of service use or cost savings for the health systems. Furthermore, transfer rates stratified by acuity of presentation and their impact on local hospital admission and ED discharge rates are also a meaningful service use measure for emergency telehealth service evaluation. This also indicates the effectiveness of specialist consultation in changing resource use patterns at a local level.

The synthesis from this review indicated that favorable health service use patterns can be expected across different clinical areas where the impact of a specialist workforce shortage is modifiable by telehealth implementation in these settings. Dorsey et al [40] observed 3 interlinking trends shaping telehealth, including the transformation from increasing access to eventually reducing cost rationalizing the potential for telehealth to reduce time spent accessing specialist services and increase the intensity of services to the 20% of the people accounting for 80% of the health expenditure. Changes in cost were not considered in this review, and the synthesis in this systematic review did not find conclusive evidence to support this trend in the context of acute presentation to rural and remote EDs. However, the increasing local hospital admission rate due to the addition of clinical expertise through emergency telehealth has been discussed in a study as a means of increasing local hospital revenue [20]. The changes in patient disposition, such as reduction in admissions and transfers [19], also align with the changes in the cost profile of the overall service delivery.

#### Patient Outcomes

Studies reporting clinical effectiveness demonstrated improved clinical effectiveness in stroke, pediatrics, burns, mental health, and ophthalmology, albeit by using different clinical or patient outcome measures. We have identified a hierarchy of outcomes in the studies: treatment-related timeliness, diagnosis accuracy, quality of consults, and treatment appropriateness are all categories of outcomes with potential impact on patients’ functional outcome or survival.

This review was conducted as part of a larger study on the cost-effectiveness of telehealth in rural and remote EDs [21]. Patient outcomes are the foundation of cost-effectiveness analyses in rural and remote EDs. The ideal outcome measure to accommodate the wide range of ED presentations is quality-adjusted life years (QALYs). When direct data collection on QALYs is not a pragmatic option, it is often calculated from a patient outcome measure. In telestroke studies, for example, QALYs are derived from the modified Rankin Scale scores. A further extension to this review on effectiveness measures in rural and remote emergency telehealth services is the question of using mortality and patient outcome measures to derive QALYs for economic evaluation. This calls for future research into the relationship between diagnosis accuracy and mortality and functional outcomes and the derivation of QALYs from the appropriate patient outcome measures for rural and remote EDs and the receiving-end (local) hospital context of the emergency telehealth service.

### Limitations

Timeliness of the clinical decision and, where appropriate, clinical intervention is critical to the effectiveness of acute ED care. In this review, 2 components that contribute to timeliness were not considered: the impact of prehospital emergency medical services and the distance factor. The decision to exclude studies on prehospital emergency medical services was made to restrict the scope of this review to the effectiveness of emergency telehealth services delivered in hospital settings.

The second limitation is related to the distance between the EDs and the destination of transfer. This, combined with the appropriateness of interhospital transfer decisions, contributes to treatment delays in transit and may affect further patient care (clinical) decisions. We were unable to determine the extent to which telehealth was effective in bridging this gap across clinical areas from the evidence reviewed. Although most studies reported on the distance between spoke and hub hospitals, it was not possible to synthesize the impact of distance on the effectiveness findings using the available information. The small number of spokes included in the studies also made it difficult to make meaningful comparisons in relation to the distance in the studies.

The heterogeneity of the studies in this field rendered the observation of relationships among different levels in the outcomes hierarchy an impossible task. However, based on the findings in this systematic review, future studies or evaluation efforts are well placed to consider an outcomes hierarchy, with surrogate outcomes leading to changes in patients’ functional outcome and mortality in the same study.

### Comparison With Prior Work

This review is the first in rural and remote emergency telehealth to demonstrate the importance of understanding the context in which effectiveness measures are applied in evaluating telehealth. In designing telehealth services in rural and remote EDs, the purpose of telehealth by clinical area and acuity of presentation should be ascertained before setting targets for the telehealth services or program. The context around the benefit of telehealth in supporting more informed clinical decisions and accurate diagnosis, more favorable health service use patterns, and longer-term patient outcomes also has policy implications.

Policy makers should be cognizant of the complexities around, and the limitations of, emergency telehealth in rural and remote settings so as to set reasonable expectations regarding the expected outcomes from this modality of service delivery. The ascertainment of service goals should commence by examining the purpose of telehealth by clinical area, acuity of presentation, receiving-end hospital capability, and the level of definitive care to set appropriate performance targets.

### Conclusions

Ascertaining outcome measures to accurately reflect the contribution of telehealth in rural and remote EDs is a complex task. Emergency telehealth studies typically use multiple outcome measures to determine the effectiveness of the services. The analysis in this systematic review has revealed 3 criteria in outcome determination in this context: clinical area, acuity of presentation, and the level of definitive care relative to the hospital of origin. These criteria are useful when defining the favorable direction for each outcome measure of interest.

The findings from this review inform the motivation and expectation of emergency rural and remote telehealth services in the design phase. The evidence from this review indicates that emergency telehealth service adoption has resulted in better service use patterns by improving the diagnosis and making first-line management modifiable by bringing in specialist expertise in emergency medicine. However, the factors that influence clinical decisions but are not modifiable by emergency telehealth, such as receiving-end hospital capability, have not been directly studied in the rural and remote ED context.

## Appendix

Multimedia Appendix 1 Summary information of the included studies.

Multimedia Appendix 2 Categorization of the included studies.

## Abbreviations

ED: emergency department
PRISMA: Preferred Reporting Items for Systematic Reviews and Meta-Analyses
QALY: quality-adjusted life year
