# Supplementary material for: Effectiveness of Telehealth in Rural and Remote Emergency Departments: Systematic Review
Source: J Med Internet Res. 2021 Nov 26;23(11):e30632. doi: 10.2196/30632 (PMC8665379; doi:10.2196/30632)
Supplement: Multimedia Appendix 1 [file jmir_v23i11e30632_app1.pdf]

# Multimedia Appendix 1 Summary information of the included studies

| Author | Year | Title | Country | Clinical Area | Reporting Quality | Aim | Objective | Telehealth Intervention | Operational Use of Telehealth | Study Type | Comparisons | Statistical Method | Data Sources | Data Collection Period | Conclusion |
|---|---|---|---|---|---|---|---|---|---|---|---|---|---|---|---|
| Saffle et al | 2009 | Telemedicine evaluation of acute burns is accurate and cost-effective | USA | Burns | 72.22% | To evaluate the feasibility and value of telemedicine-based evaluation of acutely burned patients | To compare consults and referrals from three regional hospitals over a 2 year period before and after the instigation of telehealth | Telemedicine (videoconferencing) | Specialist support | Cohort | Before and after | Two-sample t-test (non-parametric)<br>Descriptive analytics: means with interquartile ranges | 1. Project data collection<br>2. Satisfaction survey | 1 Jul 2005 to 31 Aug 2007 | Use of telemedicine facilitate delivery of care to patients with burn injries of all sizes. From the following evidence, the authors concluded that telemedicine expertise was extended to remote facilities at essentially no cost to patients.<br>* For major burns telemedicine helped to speed provision of appropriate critical care and justify the expense and ris of air transport. Lesser burns quickly identified for economical ground transport or given definitive local care at grea cost savings, all without apparent in increase in undertriage (transferred TELE-AIR patients had larger burns than PRE-TELE patients, coupled with the very small burn sizes among the other TELE patients).<br>* Telemedicine evaluation for burn injuries by experienced physicians is more accurate, and correlate more closely with live assesment than the estimates of local physicians (burn centre physician estimates of burn size made by telemedicine and direct inspection correlated closely. In contrast, burn size estimates between burn center and referring physicians are more variable; between local physician and burn center physicia using telemedicine were significantly different).<br>* Telemedicine evaluation is accurate enough to use in making decisions about triage. (Median length of stay at the burn center increased in the TELE group. Proportion requiring surgery and mean number of surgeries per patient wa not difference.)<br>* Providers and patients found the telemedicine experience helpful, comfortable with technology involved, felt enhanced local delivery of care. |
| Kleinrok et al | 2014 | Electrocardiogram teletransmission and teleconsultation: Essential elements of the organisation of medical care for patients with ST segment elevation myocardial infarction: A single centre experience | Poland | Cardio-vascular | 56.25% | To summarise several years of experience with the use of ECG teletransmission and teleconsultation system in a Polish rural region, present conclusions drawn from practical use of the suggested management algorithm, and compare effectiveness of this sytem in its early and established phase. | To analyse the size of specific components of the overall treatment delay with particular focus on those referred to in the current guidelines.<br>To present and discuss a complete and clinically established STEMI management protocol from the occurrence of chest pain to revascularisation of an epicardial coronary artery. | Direct ECG teletransmission to coronary care unit in PCI-capable hospitals from EMS stations | Diagnostics | Cohort | Early v mature telemedicine phases | Mean and standardeviation; Mann-Whitney U test using Chi square test | Prospective data collections: 1. questionnaired filled by person in charge of EMS together with the patient to document pre-hospital delay. 2. In-phospital delay from standard hospital medical records. | 2006 (first successful ECG teletransmission on October 6, 2005) and 2010 (after data stablisation) | ECG teletransmission and teleconsultation system reduces the system delay (8.3% reduction in system delay, 24% in hospital delay; 118.6% increase in primary percutaneous coronary intervention performed within 90 min after first medical contact (FMC). 32.1% decrease in FMC to diagnosis. No change in patient-related delay. |
| Southard et al | 2014 | Telemental health evaluations enahance access and efficiency in a critical access hospital emergency department | USA | Mental Health | 68.75% | To examine the effectiveness of an emergency telemental health evaluation service implemented in rural hospital emergency room. | To examine the avearge length of time it took for patients to receive emergency mental health evaluations.<br>To examine the avearge legnth of time it took for mental health patients' subsequent release from the rural hospital after implementation of telemedicine program.<br>To examine changes in rural hospital emergency department wait time for patient for whom mental healht evaluations were ordered. | Telemedicine (did not specify VC or telephone) | Direct consultation | Cohort | Before and after | Two-sample t-test | Restrospective data collection from hospital charts of a naturally occuring serial patient sample. | 1 Jan 2009 to 31 Jan 2010 | Using telemedicine made mental health evaluations readily available around the clock in areas where such services would not otherwise be available as evidenced by:<br>* Average length of time for patients to receive emergency mental health evaluations and to subseqeuntly be released from the rural hospital was significantly lower after implementation of the telemedicine program.<br>* When 24/7 access became available, distribution of triage and consultations shifted from day to primarily evenings and nights.<br>* Increased number of disposition options safely and practically considered by ER providers when consultations made via telemedicine (home with follow-up, tertiary care center, behavioural facility in addition to inpatient observation, the only option available before telemedicine). |
| Vakkalanka et al | 2019 | Telemedicine is associated with rapid transfer and fewer involuntary holds among patients presenting with suicidal ideation in rural hospitals: a propensity matched cohort study | USA | Mental Health | 90.91% | To evaluate the impact of mental health specialty telemedicine consultation on clinical management and outcomes of patients seen for suicidal ideation/attempt in critical access hospital EDs across a rural telemedicine netowrk. | To measure the association between telemedicine consultation and ED length of stay, with the hypothesis that those who had telemedicine incorporated would spend less time in rural EDs.<br>To measure the association between telemedicine consultation and a series of secondary outcomes. | Telemedicine (did not specify VC or telephone | Specialist support | Cohort | CS - Telemedicine v no specialist consultation | Conditional linear and logistic regression of propensity-matched pairs | 1. visit-level adminstrative claims data; 2. telemedicine provider log file to indicate telemedicine exposure; 3. manual chart review of hospital medical records for clinical histories, medications and outcomes; 4. National Death Index | 1 Nov 2007 to 30 Sep 2015 | Telemedicine ED LOS was associated with a 29.3% decrease in transferred patients, 2.35 times greater odds of hospital admission (local or through transfer), and lower involuntary hold placement (OR 0.48). |
| Benger et al | 2004 | The safety and effectiveness of minor injuries telemedicine | UK | Minor Injury | 100% | To determine the safety of minor injuries telemedicine compared with on-site specialist care, current practice, and a robust gold standard, and to assess the clinical effectiveness of this new technique. | To directly compare three indepednent medical assessments with a gold standard.<br>To conduct a prospective, randomised, and blinded trial to measure clinical effectiveness and collect process measures relating to consultation duration, radiograph requests, and follow up. | Telemedicine (videoconferencing) | Direct consultation | RCT | CS - Telmedicine v face to face | Cochran Q test and McNemar's Chi Square test; paired t test; Chi square test; Kruskal-Wallis equality of population rank test. | Safety against Gold standard: as assessed by a panel of 10 emergency medical consultants. Scores allocated used to calculate mean and median discrepancy score.<br>Process outcomes from hospital data collection.<br>Clinical effectiveness using 6 item checklist (Box 3) | December 1999 to November 2000 | Telemedicine is capable of providing a satisfactory standard of care: safety of minor injuries telemedicine is similar to conventional practice. No significant difference between the errors for onsite emergency specialist assessment, telemedicine assessment, and in person GP assessment.<br>Telemedicine requires twice as long as an onsite consultation, in addition to the time entailed in responding to a request and breaking away from other activities. |
| Bowman et al | 2003 | Reliability of telemedicine for diagnosing and managing eye problems in accident and emergency departments | UK | Ophthalmology | 100% | To test the reliability of telemedicine for diagnosis and management in an opthalmic accident and emergency setting. | To measure the clinical agreement between two senior opthalmological residents, comparing live/live consultations with live/telemedicine consultations.<br>To assess the clinical agreement using only telemedicine (eg videoconference with torchlight and no slit lamp) in addition to that using a video slit lamp. | Telemedicine (videoconferencing) | Specialist support | Non-randomised Trial | CS - Telmedicine v face to face | Chi-square | Trial data collection | Did not document | "Telemedicine utilising video slit lamp images was found to be a safe and reliable method of diagnosing and managing eye problems presenting to accident and emergency departments. Telemedicine did have more clinical disagreements, but these erred on the side of caution, implying that ophthalmic problems in general accident and emergency departments could be managed using videoconferencing for secondary advice from an ophthalmologist. Potentially this offers a cost-effective way of providing ophthalmic support and advice to smaller hospitals and general practices which form part of an integrated service network." (p746) |
| Dharmar et al | 2013 | Telemedicine consultations and medication errors in rural emergency departments | USA | Paediatrics | 100.00% | To evaluate the frequency of medication errors in rural emergency department telemedicine consultations. | To compare the frequency of physician-related medication errors among seriously ill and injured children receiving telemedicine consultations, telephone consultations, no consultation in rural EDs. | Telemedicine (videoconferencing) or Telephone | Specialist support | Cohort | CS - Telmedicine v telephone or no specialist consultation | Hierarchical logistic regression adjusted for covariates (age, risk of admission, year of consultation, hospital) | Retrospective chart audit by two pediatric pharmacists to identify and categorise medication errors using medication error instrument.<br>File audit to extract context variables. | 1 Jan 2003 to 31 December 2009 | "The use of telemedicine to provide pediatric critical care consultations to rural EDs was associated with significantly fewer physician-related medication errors than providing critical care consultations by telephone or providing care to similarly ill children without pediatric critical care consultations." (p1095) |
| Harvey et al | 2017 | The Impact of Telemedicine on Pediatric Critical Care Triage | USA | Paediatrics | 100.00% | To evaluate the impact of pediatric critical care telemedicine consultation on triage decision making and to evaluate the consulting intensivists' perspective on the accuracy and benefits of the telemedicine consultation. | To examine the relationship between pediatric critical care telemedicine consultation in rural emergency departments and triage decisions. Triage locaiton and provider ating of the accuracy of remote assessment for a cohort ofp atients who received critical care telemedicine consultations and a similar group of patients receiving telephone consultations will be compared. | Telemedicine (videoconferencing) | Specialist support | Cohort | CS - Telemedicine v telephone | Chi-square to examine relationship; Linear regression model using binomial distribution and a logit link to predict triage location as a function of telemedicine consult age and sex. | Project patient registry collected by MUSC consulting provider using a REDCap survey.<br>Subjective accuracy assessment of telemedicine and telephone consultation process, decision making, quality and perceived accuracy of assessment, and outcomes. | 1 April 012 to 31 March 2016 | Pediatric cricial care telemedicien consultation in community hospitals results in a significant reduction in PICU admission. Critical care providers in one academic hospital viewed telemedicine as being more accurate than telephone consutations and believed telemedicine impacted their triage decision. |
| Westbrook et al | 2008 | Impact of an ultrabroadband emergency department telemedicine system on the care of acutely ill patients and clinicians' work | Australia | Rural and Remote ED | 100.00% | To evaluate whether the introduction of an ED telemedicine system change patient management and outcome indicators and to investigate clinicians' perceptions of the impact of the system on care provided and on their work. | To compare patient management and patient outcome indicator data before and after the ViCCU intervention.<br>To conduct interviews with clinicians (doctors and nurses) at the two sites. | Virtual Critical Care Unit (ViCCU) - videoconferencing | Specialist support | Cohort | Before and after | Chi-square tet for case type, age, sex; medians and median tests for treament times and length of stay comparisons; logistic regression for each case stype to test for confounding. | 1. Extracted from administrative data<br>2. Semi-structured interviews | 1 Jan to 31 Dec 2003 (BV)<br>1 Jan 2004 to 30 June 2005 (AV) | ViCCU most effective for moderate trauma patients with 45-63% increase in discharge and 48-25% decrease in transfers. |
| Mueller et al | 2014 | Lessons from Tele-emergency: Improving care quality and health outcomes by expanding support for rural care systems | USA | Rural and Remote ED | 30% | To evaluate the 24/7 tele-emergency service in the upper Midwest connecting a hub ED and 71 remote hospitals. | To identify three tele-emergency models and associated outcomes.<br>To describe findings from evaluation including user survey and interviews with clinicians and administrators. | Tele-emergency (audi or video) | Specialist support | Qualitative | Before and after | Simple proportion for surveys. Nil for qualitative analysis. | 1. Clinician and administrator survey; 2. Telephone interviews<br>3. Onsite interviews | 2011 phoe interviews;<br>Feb-Apr 2013 surveys;<br>2013 onsite interviews | Participants reported improved clinical quality, expanded care team, increased resources during critical events, shortens time to care, improves care coordination, promotes tablizes the rural hospital patient base. Inconsistent reimbursement policy, cross-state licensing barriers and other regulations hinder tele-emergency implementation. |
| Sterling et al | 2017 | The Impact of the TelEmergency (TE) program on rural emergency care: An implementation study | USA | Rural and Remote ED | 80.00% | To evaluate the effect of the TE program on rural hospital ED operations, and to evaluate the satisfaction of the NPs in the TE program. | To exam,ine how the implementation of a NP and EM physician model impact rural hospital operation.<br>To determine NP utilisation and satisfaction with the TE system. | TelEmergency program (videoconferencing) | Direct consultation | Cohort | Before and after | Descriptive statistics, Wilcoxon signed rank test, nonparametric paired t-tests. | 1. Project data collection on ED and hospital operation<br>2. 21-question hospital administrator and NP survey administered 24 months following implementation<br>3. Telemedicine ED registrations; medicare cost reports, hospital admission registration data.<br>4. Electronic health record available only after TE implementation | Jan-Dec 2007 (before TE)<br>Jan-Dec 2008 (after TE) | Significant decrease in discharge rate from ED, increase in rural hospital admission rate from ED, and discharge against medical advice from ED also increased. |
| Mohr et al RR | 2018 | Emergency Department Telemedicine Shortens Rural Time-To-Provider and Emergency Department Transfer Times | USA | Rural and Remote ED | 100.00% | To measure the impact of ED-based telemedicine on timeliness of care in participating rural hospitals | To measure the impact of ED-based telemedicine services on door-to-provider time in a single telemedicine network in the upper Midwest.<br>To estimate the effect on ED length of stay (LOS) and time to transfer. | Telemedicine (videoconferencing) | Direct consultation | Cohort | Before and after | Wilcoson rank-sum test for continuous variables; chi square for categorical variables | Routine administrative data collection | 2007 to 2015 | Telemedicine decreases ED door-toprovider time, most commonly because the telemedicine provider was the first provider seeing a patient. Among transferred patients, ED length of stay at the first hospital was shorter in patients who had telemedicine consulted. |
| Wang et al | 2000 | Treating Acute Stroke Patients With Intravenous tPA The OSF Stroke Network Experience | USA | Stroke | 54.55% | To examine the safety and outcome of intravenous tPA for acute ischemic stroke in the OSF Stroke Network. | To present the experience of treating 57 acute ischemic stroke patients among the OSF SN hospitals | Consultation with a neurologist (did not specify telephone or videoconferencing) | Specialist support | Cohort | CS - Hub v spoke | Descriptive and frequency statistical analyses | Demographic information obtained from medical records.<br>Project variable collection. | June 1996 to December 1998 | tPA can be safely and effectively given with good outcome in patients with acue ishcemic stroke |
| Meyer et al | 2008 | Efficacy of site-independent telemedicine in the STRokE DOC trial: a randomised, blinded, prospective study | USA | Stroke | 100% | To prospectively assess whether telemedicine (real-time, 2 way audio/video, and DICOM interpretation) or telephone was superior for decision-making in acute telmedicine consultations | To test the hypothesis that telemedicine increases decision-making efficacy / to determine the efficacy of telemedicine consultations for decision-making. | Telemedicine (videoconferencing) | Direct consultation | RCT | CS - Telemedicine v telephone | Cochran-Mantel-Haenszel (CMH) chi-squared test, stratified by site, and fixed-effect logistic regression were conducted as a sensitivity analysis. The CMH chisquared test, stratified by site, and fixed-effect logistic regression were used for all other 'correct decision' outcomes. The Fisher's Exact test was used for rt-PA rate, ICH rate, mortality, 90 day mRS and missing data analysis. The Wilcoxon Rank Sum was used for 90 day Barthel and time-point comparisons. | The STRokE dOC Adjudicating Committee (SDAC) composed of specialist physicians with training in acute stroke, and excluded practitioners from the remote spoke facilities: determine correctness of rt-PA decision. | Aug 2004 to April 2007 | This trial reports that stroke telemedicine consultations result in more accurate decision making compared to telephone (98.2% telemedicine made correct decisions, 82% telephone made correct decision (P=0.0009), and can serve as a model for the effective use of telemedicine in other medical fields. The more appropriate decisions, high PA userates (28% telemedicine, 23% telephone (p=0.4248)), improved data collection (3.1% telemedicine incomplete data, 12% telephone incomplete data); no difference in post rt-PA ICH rates; low technical complications, and favorable time requirements all support telemedicine's efficacy, most specifically for decision-making, and may enable more practitioners to use telemedicine in daily stroke care. |
| Sairanen et al | 2011 | Two years of Finnish Telestroke: Thrombolysis at spokes equal to that at the hub | Finland | Stroke | 72.73% | To describe the characteristics and 3-month outcome of the thrombolysis patients treated in 5 community hospitals served by the Helsinki University Central Hospital (HUCH) in a telestroke network during 2007 to 2009 | To compare the the demographic characteristics, NIHSS before thrombolysis (i.e. baseline NIHSS), and onset to threatment time in patient groups who did or did not receive thrombolysis. | TeleStroke (videoconferencing) | Specialist support | Cohort | Telemedicine and population | Student t test or Mann-Whitney rank sum test for continuous variables, Pearson Chi Square test for discrete variables. | Finnish TeleStroke pilot project data for the intervention group; Helsinki Stroke Thrombolysis Registry for the comparison group. | May 2007 to May 2009 from the Finnish TeleStroke project; the Helsinki Stroke Thrombolysis Registry contains cases from 1998 to 2008 | Telestroke thrombolysis results (onset to treatment time, safety, and efficacy) are comparable to those achieved in dedicated thrombolysis center.<br>The Finnish Telestroke Network led to an exceptionally high thrombolysis rate of 57.5% of the consultations with no significant difference among the proportion in the Finnish spokes compared with 15.5% and 29.8% in Candian and Germany projects.<br>Three month outcome of patients treated with thrombolysis was equally favourable with comparable rates of symptomatic intracerebral bleedings between the Finnish telestorke spokes and at an experienced hub (HUCH) |
| Yaghi et al | 2015 | Post t-PA transfer to hub improves outcome of moderate to severe ischemic stroke patients | USA | Stroke | 90.91% | To determine whether transfer of patients to hub hospital is beneficial. | To compare the outcome of patients who were transferred to a 'hub' with those who remained at the 'spoke' hospital where thrombolysis took place. | Telemedicine (VC and teleradiology) | Specialist support | Cohort | CS - Hub v spoke | Fisher's exact test for gender, history of hypertension , daibetes, hyperlipidemia, atrial fibrillation, admission NIHSS score, 3 months outcome, mortality | Prospective data collection maintained by the Arkansas Stroke Assistance through Virtual Emergency Support (AR SAVES); outcome obtained through phone interview by a certified nurse coordinator with the patient or caregiver. | November 2008 to January 2012 | The subgroup of patients with moderate to severe stroke (NIHSS >8) benefited significantly from transfer to a primary stroke centre (PSC) designated hub hospital. The availability of stroke neurologist, neuro-intensivists, interventional neurologist, better stroke education and secondary prevention comprise some of the resources unique to a PSC; however, the authors believe stroke quality measures including prompt initiation of antiplatelet agents, anticoagulants and lipid lowering agents for stroke prevention are some important considerations. |
| Barlinn et al | 2017 | Acute endovascular treatment delivery to ischemic stroke patients transferred within a telestroke network: a retrospective observational study | Germany | Stroke | 90.91% | To define the characteristics of patients undergoing EVST for acute intracranial large vessel occlusion after transferal to a tertiary care stroke center via a hub and spoke telestroke network | To compare clinical outcomes of EVST between anterior circulation stroke patients transferred after teleconsultation and those directyly admitted to a tertiary stroke centre | Telemedicine (VC and teleradiology) | Specialist support | Cohort | CS - Hub v spoke | Median (interuartile range) and percentage for continuous and non-continuous variable.<br>Chi square, Fisher's exact test, Mann-Whitney U test for study group comparisons.<br>Multivariate logistic regression to explore predictive value of telestroke for sICH, in-hospital death, reperfusion status, favourable functional outcome. | Prospectively maintained databse of patients who were emergenl treated with EVT. | January 2010 to December 2014 | "Telestroke networks may enable delivery of EVT to selected ischemic stroke patients transferred from remote hospitals that is equitable to patients admitted directly to tertiary hospitals." (p508) |

| Author | Year | Title | Country | Clinical Area | Reporting Quality | Aim | Objective | Telehealth Intervention | Operational Use of Telehealth | Study Type | Comparisons | Statistical Method | Data Sources | Data Collection Period | Conclusion |
|---|---|---|---|---|---|---|---|---|---|---|---|---|---|---|---|
| Bladin et al | 2020 | Improving acute stroke care in regional hospitals: clinical evaluation of the Victorian Stroke Telemedicine program | Australia | Stroke | 100.00% | To evaluate the impact of the VST program during its first 12 months on the quality of care provided to patients presenting with suspected stroke to participating hospitals in regional Victoria. | (same as aim) | Telemedicine (VC and teleradiology) | Specialist support | Cohort | Before and after | Chi-square test for categorical variable; Kruskal-Wallis test for continuous variables; Logistic regression for association of telemedicine with patient treatment and outcome (ischaemic stroke only); Median regression for association of telemedicine with time to diagnosis and treatment (ischaemic stroke only)<br>All models adjusted for age, sex, history of stroke, ability to walk on admission, hospital size, patient clustering by hospital | Prospectively collected data collection by site staff | intervention period (12 months during 8 January 2014 to 31 December 2017)<br><br>control period (12 months preceding implementaiton) | "Telemedicine has provided Victorian regional hospitals access to expert care for emergency department patients with suspected acute stroke. Eligible patients with ischaemic stroke are now receiving stroke thrombolysis more quickly and safely" (abstract)<br>"A slightly larger proportion of patients with ischaemic stroke who arrived within 4.5 hours of symptom onset received thrombolysis during the intervention than during the control period (37% v 30%). Door- to- CT scan time (median, 25 min [IQR, 13–49 min] v 34 min [IQR, 18–76 min]) and door- to- needle time for stroke thrombolysis (73 min [IQR, 56–96 min] v 102 min [IQR, 77–128 min]) were shorter during the intervention. The proportions of patients who received thrombolysis and had a symptomatic intracerebral haemorrhage (4% v 16%) or died in hospital (6% v 20%) were smaller during the intervention period."(abstract) |
| Duchesne et al | 2008 | Impact of telemedicine upon rural trauma care | USA | Trauma | 80.00% | To evaluate the impact of telemedicine upon rural trauma care. | To analyse outcome before (pre-TM) and after (post-TM) implementation of telemedicine in the management of rural trauma patients initially treated at local community hospitals before trauma centre transfer. | Telemedicine (videoconferencing) | Direct consultation | Cohort | Before and after | Two-sample t-test or Chi-square analysis | Retrospective data collection 2.5 years before telemedicine implementation, 2.5 years post telemedicine implementation. | January 2000 to January 2005 | "Telemedicine significantly improved rural LCH evaluation and management of trauma patients. More severely injured trauma patients were identified (less blunt trauma, higher ISS, initial base deficit, more units of packed red cell transfusion needed among patients transferred to TC post-TM) and more rapidly transferred to the TC. Total TC hospital costs were significantly decreased without significant changes in TC mortality. Introduction of telemedicine consultation to rural LCH emergency departments expanded LCH trauma capabilities and conserved TC resources, which were directed to more severely injured patients." (abstract) There was a significant decreases in the LOS at th LCH, transfer time from LCH to the TC and TC hospital cost. |
| Bolle et al | 2009 | Video conferencing versus telephone calls for team work across hospitals: a qualitative study on simulated emergencies | Norway | Trauma | 60% | To study if VC could improve communication and team function between rural and central emergency hospital teams with several participants at either side of the video link. | To study how the teams cooperated during simulated complex trauma and emergency medicine scenarios, and compared the strength and weaknesses of VC with standard telephone communication. | Telemedicine (videoconferencing) | Direct consultation | Qualitative | CS - Telemedicine v telephone | Nil (qualitative analysis) | External observer observations of each of the 9 simulated emergency scenarios focusing on intra and inter-group communication. Semi-structured group interviews following each scenario. | Did not document | VC improve communication for treating and triaging emergency patients by increasing interactions, increase likelihood of gainig a common understanding and support simultaneous work. Increased team size cause interruptions to work flow. VC cut time before patient is seen by specialists and may positively affect outcome. |
| Mohr et al T | 2018 | Telemedicine use decreases rural emergency department length of stay for transferred North Dakota trauma patients | USA | Trauma | 100.00% | To evaluate the impact of a rural telemedicine network on trauma process of care and cinical outcomes in North Dakota | To measure the association between telemedicine use and ED length of stay (LOS), with secondary outcomes of X-ray and computed tomography (CT) utilisation, trauma procedures, and mortality. | Telemedicine (videoconferencing) | Direct consultation | Cohort | CS - Telemedicine v no specialist consultation | Proportional and mean difference for characteristics and primary outcome comparisons. Adjusted Odds Ratio (aOR) for secondary outcomes. | North Dakota Trauma Registry (NDTR) | 2008 to 2014 | Telemedicine use shortens ED LOS for transferred patients, but has little effect on other process of care metrics for rural trauma patients in critical access hospitals. Telemedicine availability was associated with increased CT use, X-ray use, and proportion of interhospital transfers. Among rural North Dakota trauma patients, telemedicine was not associated with mortality. |
