# Supplementary material for: Effectiveness of Telehealth in Rural and Remote Emergency Departments: Systematic Review
Source: J Med Internet Res. 2021 Nov 26;23(11):e30632. doi: 10.2196/30632 (PMC8665379; doi:10.2196/30632)
Supplement: Multimedia Appendix 2 [file jmir_v23i11e30632_app2.pdf]

Table S1 Study categories: clinical area by country of telehealth implementation

Clinical Area	Australia	Finland	Germany	Norway	Poland	UK	USA	Total
**Burns**							1	1
**Cardio-vascular**					1			1
**Mental Health**							2	2
**Minor Injury**						1		1
**Ophthalmology**						1		1
**Paediatrics**							2	2
**Rural and Remote ED**	1						3	3
**Stroke**	1	1	1				3	6
**Trauma**				1			2	3
**Total**	**2**	**1**	**1**	**1**	**1**	**2**	**13**	**21**

Table S2 Study categories: clinical area by operational use of telehealth

Clinical Area	Diagnostics	Direct consultation	Specialist support	Specialty Total
**Burns**			1	1
**Cardiovascular**	1			1
**Mental Health**		1	1	2
**Minor Injury**		1		1
**Ophthalmology**			1	1
**Paediatrics**			2	2
**Rural and Remote ED**		2	2	4
**Stroke**		1	5	6
**Trauma**		3		3
**Operational Use Total**	**1**	**8**	**12**	**21**

Table S3 Study design by comparison categories of included studies

Comparison Type	Experimental	Cohort studies	Qualitative	Comparison Subtotal
**Before and after**		Duchesne et al (2008)<br>Mohr, Young et al (2018)<br>Saffle et al (2009)<br>Southard et al (2017)<br>Sterling et al (2017)<br>Westbrook et al (2008)	Muller et al (2014)	7
**Cross section comparison**	**Non-randomised**<br>Bowman et al (2003)<br>**Randomised**<br>Benger et al (2004)<br>Myer et al (2008)	Barlinn et al (2017)<br>Dharmar et al (2013)<br>Harvey et al (2017)<br>Mohr, Vakkalanka et al (2018)<br>Vakkalanka et al (2019)<br>Wang et al (2000)<br>Yaghi et al (2015)	Bolle et al (2009)	11
**Early v mature telemedicine phases**		Kleinrok et al (2014)<br>Bladin et al (2020)		2
**Telemedicine and population**		Sairanen et al (2011)		1
**Study Design Subtotal**	3	16	2	21
